# Hydroalcoholic extract from *Origanum vulgare* induces a combined anti-mycobacterial and anti-inflammatory response in innate immune cells

**DOI:** 10.1371/journal.pone.0213150

**Published:** 2019-03-04

**Authors:** Federica De Santis, Noemi Poerio, Angelo Gismondi, Valentina Nanni, Gabriele Di Marco, Roberto Nisini, Maria Cristina Thaller, Antonella Canini, Maurizio Fraziano

**Affiliations:** 1 Department of Biology, University of Rome “Tor Vergata”, Rome, Italy; 2 Department of infectious diseases, Istituto Superiore di Sanità, Rome, Italy; Institut de Pharmacologie et de Biologie Structurale, FRANCE

## Abstract

In nature, many plants or their extracted compounds have been found to possess anti-inflammatory features and therapeutic properties against infectious as well as non-infectious diseases, including cancer. In this study, we analysed the immunomodulatory effects on innate immune cells of hydroalcoholic extract from *Origanum vulgare* L. ssp. *hirtum* (HyE-Ov), a plant traditionally known for its anti-oxidative properties. The effects of HyE-Ov were tested on human monocyte derived dendritic cells (DC), type-1 (M1) and type-2 macrophages (M2) infected with *M*. *bovis* Bacille Calmette-Guérin (BCG), used as a model of persistent intracellular bacterium. DC, M1 and M2 treated with HyE-Ov significantly enhanced their mycobactericidal activity, which was associated with phagosomal acidification in M1 and M2 and increase of phagosomal, but not mitochondrial ROS production in M1, M2, and DC. Treatment of BCG-infected DC with HyE-Ov significantly reduced TNF-α and IL-12 production and increased TGF-β synthesis. Finally, experiments were repeated using eight different HPLC fractions of HyE-Ov. Results showed that the capability to activate anti-microbial and anti-inflammatory response is shared by different fractions, suggesting that diverse bioactive molecules are present within the hydroalcoholic extract. Altogether, these results show that HyE-Ov promotes anti-mycobacterial innate immunity and limits inflammatory response *in vitro* and suggest that this plant extract may be exploitable as phytocomplex or nutraceutical for novel host-directed therapeutic approaches.

## Introduction

Since ancient times, man has used plants to obtain therapeutic benefits and there is now an increasing scientific interest for their biological properties as they can provide a plethora of novel molecules of pharmacological interest [1]. An assessment of all Food and Drug Administration (FDA)- and European Medicine Agency (EMA)-approved molecules reveals that natural products and their derivatives represent over one-third of all new molecules, with one quarter of them derived from plants [2] as they may contain a variety of biologically active secondary metabolites with possible therapeutic value against infectious as well as non-infectious diseases and cancer [1, 3–5]. Among plants of potential medical interest, *O*. *vulgare* L. is known for its properties as expectorant, antimicrobial and carminative [6]. *Origanum* genus belongs to the Lamiaceae family and most of its species are distributed around the Mediterranean area, Eurasia and the North of Africa, where it is used in traditional medicine for the treatment of cold, cough, digestive and respiratory disorders [6, 7].

The therapeutic effect of oregano in traditional medicine was mainly attributed to the antimicrobial, anti-inflammatory and antioxidant properties shown by its phenolic compounds [8–10]. This biological effect is maintained in methanol and ethanol macerations, infusion, decoction, hydroalcoholic extract, other than in essential oils [11–16]. In particular, anti-inflammatory activity of *O*. *vulgare* is due to the capability of its extracts to reduce production of nitric oxide, to decrease and increase the production of inflammatory and anti-inflammatory cytokines, respectively [17–20]. In addition, a direct antimicrobial activity has been reported against fungi [21, 22], gram-positive and gram-negative bacteria [13, 14, 23]. This effect was induced by the phenolic monoterpenes, thymol and carvacrol, which together with their precursors *p*-cymene and γ-terpinene constitute the main component (about 80%) of the essential oils of this plant [13]. The intensity of antibacterial activity is directly related to the amount of these compounds, which in turn strictly depends on geographical origins, climatic growing condition, soil constitution, harvesting period, extraction method and the target bacterial species [21, 24].

Tuberculosis (TB) is an infectious disease caused by *Mycobacterium tuberculosis* (MTB) and still represents one of the main causes of death by single pathogen infection, worldwide. According to the last report by World Health Organization (WHO), in 2017, 10.0 million people fell ill with TB and 1.6 millions of them died [25]. Today, the emergence of mycobacterial strains, pathogenic to humans, endowed with resistance to first-line (Multi-Drug Resistant, MDR) and second-line antibiotics (Extensively-Drug Resistant, XDR) represents an important global problem. WHO estimated 558.000 MDR-TB cases, 8.5% of which were characterized by XDR-TB [25]. The emergence of MDR and XDR mycobacterial strains has led to the need to define new therapeutic options that, in association with standard chemotherapies, may enhance their effectiveness and represent an additional tool for the control of drug resistance [26]. In this context, the use of adjunctive host-directed therapies (HDTs), which aim to simultaneously limit inflammation and pulmonary damage and boost the host innate antimicrobial response, may represent an attractive avenue deserving further research [26, 27].

Aim of this work was to evaluate the immunomodulatory properties of oregano plant in an *in vitro* mycobacterial infection model by using primary human dendritic cells and type-1 and type-2 macrophages, considered primary targets of *M*. *tuberculosis* infection. In particular, the immunotherapeutic value of hydroalcoholic extract of *O*. *vulgare* has been assessed in terms of activation of intracellular bactericidal machinery and of down-regulation of potentially tissue damaging inflammatory response following *in vitro* infection with *Mycobacterium bovis* Bacille Calmette-Guérin (BCG).

## Materials and methods

### Bacteria

*Mycobacterium bovis* BCG Pasteur strain (TMC1011) was grown in Middlebrook 7H9 (Difco) broth supplemented with 10% ADC (albumin, dextrose and catalase), and 0.05% Tween 80, and titred by CFU assay, performed in plates with Middlebrook 7H10 (Difco) supplemented with 10% OADC (oleic acid, albumin, dextrose and catalase), as described [28, 29]. BCG transformed with the plasmid carrying *Vibrio harveyi* luciferase gene, LuxAB, in shuttle plasmid pSMT1 (BCG-lux), kindly provided by Prof. R. Reljic from S. George’s University of London (UK), was grown and used to evaluate intracellular mycobacterial viability, as described [30]. To ensure plasmid maintenance, BCG-lux was grown by adding 50 μg/ml Hygromycin B (Invitrogen) in culture media.

### Cell cultures

Buffy coats from anonymized healthy donors, who gave their written informed consent to donate the non-clinically usable components of their blood for scientific research, were obtained from the Blood Transfusion Unit of Policlinico “Umberto I” in Rome. The present study, which is based on non-clinical *in vitro* research, did not require any specific approval from an Ethical Committee, according to the current italian law (decree by Ministero della Salute by February 8^th^, 2013, published on Gazzetta Ufficiale della Repubblica Italiana no. 96 of April 24^th^, 2013, and legislative decree no. 211 of June 24^th^, 2003, published on Gazzetta Ufficiale della Repubblica Italiana no. 184 of August 9^th^, 2003).

Peripheral blood mononuclear cells (PBMCs) were isolated by Ficoll density gradient centrifugation Monocytes were then purified by using magnetic microbeads, conjugated to monoclonal anti-human CD14 antibodies (Miltenyi Biotec), according to the manufacturer’s instructions. Monocytes were then cultured for 5 days at 37°C with 5% CO_2_ in complete medium (RPMI 1640 supplemented with 10% fetal bovine serum, 2 mM L-Glutamine and 5 μg/ml Gentamicin) in presence of either 35 ng/ml Granulocyte Macrophage-Colony Stimulating Factor (GM-CSF, Sigma-Aldrich), or 50 ng/ml Macrophage-Colony Stimulating Factor (M-CSF, R&D System), or 20 ng/ml GM-CSF plus 20 ng/ml Interleukin-4 (IL-4, Miltenyi Biotec), to get type-1 macrophages (M1), type-2 macrophages (M2), and dendritic cells (DC), respectively. Cells were then used at the density of 5x10^5^ or 10^6^/well in 24-well plates or at the density of 2x10^5^ or 3x10^5^/well in 96-well plates, as indicated in the different experiments.

### Preparation of hydroalcoholic extract of *Origanum vulgare*

Plant material (*Origanum vulgare* ssp. *hirtum*), collected at Mount Athos Vatopedi Holy Monastery in Greece during Summer 2016, was taxonomically identified through morphologic analysis. The whole plant was pounded in liquid nitrogen and incubated in hydroalcoholic solution (50% Ethanol), in agitation at room temperature for 24 hours, at a final concentration of 200 mg of fresh sample material per ml. The extract was filtrated (0.2 μm), aliquoted and completely desiccated by vacuum dry evaporating (at 30 °C), by using a Concentrator Plus (Eppendorf). Samples were stored at -80 °C until the use when they were resuspended in RPMI 1640, maintaining the original concentration of 200 mg/mL, and centrifuged, to discard the residual pellet. For treatments, 15 μl of hydroalcoholic extract of *O*. *vulgare* (HyE-Ov), corresponding to 3 mg equivalent of plant material, were used for each ml of culture medium.

### Chromatographic analysis and fractionation of HyE-Ov

HyE-Ov was subjected to a qualitative chromatographic analysis by High Performance Liquid Chromatography (HPLC) system, equipped with SPD-M20A Diode Array Detector (DAD) (Shimadzu, Japan). The investigations were carried out using a Phenomenex Luna C18(2) column (3 μm x 4.6 mm x 150 mm; 3μm), formic acid 1% (phase A) and methanol (phase B) as solvents and an elution gradient, at flow rate of 0.95 mL/min, set as follows: t0 min (A 85%, B 15%); t20 min (A 65%, B 35%); t55 min (A 10%, B 90%); t68 min (A 85%, B 15%); t70 min (A 85%, B 15%). Chromatograms were acquired by monitoring sample absorbance at 280 nm. Oregano extract fractionation was performed under the same conditions. A total of 8 fractions were separated: 7 of them were collected every 5 minutes of run (from 0 min to 35 min), whereas the eighth one consisted of the final eluate (from 35 min to the end of the run). All fractions were totally concentrated by vacuum dry evaporating (at 30 °C), using a Concentrator Plus (Eppendorf) and stored at -80 °C. For treatments, pellets were suspended in appropriate volumes of RPMI 1640, to obtain the same concentrations present in the original total extract.

### Infection with BCG or BCG-lux

BCG or BCG-lux stored at -80°C, after thawing, were centrifuged, suspended in suitable medium without Gentamicin and sonicated, in a bath sonicator, for 3 min to remove mycobacterial clumps. Afterwards DC, M1 and M2 cells were infected for 3 hours at the multiplicity of infection (MOI) of 5 or 10. After removal of extracellular bacilli, cells were stimulated or not with 3 mg/ml of HyE-Ov for 3 days or with the different HyE-Ov purified fractions, used at the same concentrations present in the original total extract. Where indicated, the following inhibitors were added: 100 U/ml polyethylene glycol-Catalase (PEG-Cat) (Sigma-Aldrich), 10 μM MitoTEMPO (Enzo Life Sciences) and 10 nM Concanamycin A (Santa Cruz Biotechnology) to block intracellular ROS, mitochondrial ROS and phagosomeacidification, respectively. Intracellular mycobacterial viability was assessed on BCG-lux infected-cells by luminometric analysis, as described [28, 30]. Briefly, cells were incubated with 0.1% saponin at 37°C for 30 min. Luminometric analysis was performed using PBS, mycobacterial suspension and 1% decanal (as luciferase substrate), at the ratio 8:1:1. Data are expressed as Replication Index, calculated as the ratio between the Relative Luminescence Units (RLU) obtained at day 3 from infection (t3) and the mean of RLU values of triplicate cultures obtained immediately after 3 hours infection (t0). Luminescence has been evaluated by the use of a Varioskan LUX Multimode Microplate reader (Thermo Fisher Scientific).

### Fluorometric analysis

Intraphagosome acidification of vacuoles containing BCG was monitored by using BCG labelled with 0.1 mg/ml of the pH sensitive dye 5(6)-Carboxyfluorescein N-hydroxysuccinimide ester (NHS) (Sigma-Aldrich), as described [28, 29, 31]. Briefly, DC, M1 and M2 cells were infected with BCG-NHS for 3 hours (MOI 5) and then stimulated with 3 mg/ml of HyE-Ov. Fluorometric analysis was performed immediately, and at 30 minutes, 120 minutes, 18 hours and 48 hours after stimulation. Intraphagosome acidification was determined as fluorescence intensity decrease, measured at an excitation wavelength of 492 nm and emission wavelength of 517 nm.

Reactive Oxygen Species (ROS) generation was analysed by labelling cells with the fluorescent indicator dichlorofluorescein (DCF, Molecular Probe). In particular, DC, M1 and M2 cells were infected with BCG (MOI 5) for 3 hours, labelled with 10 μM of DCF for 60 min at 37°C in the dark and then stimulated or not with HyE-Ov. Fluorometric analysis was performed at 20, 60, 120 minutes and 24 hours from stimulation at an excitation wavelength of 495 nm and emission wavelength of 527 nm. All data were expressed as Relative Fluorescent Units (RFU). Fluorescence has been evaluated by the use of a Varioskan LUX Multimode Microplate reader (Thermo Fisher Scientific).

### Quantification of cytokines by Enzyme-Linked Immunosorbent assay

The levels of Tumor Necrosis Factor-α (TNF-α), IL-12 and Transforming Growth Factor-β (TGF-β) in the supernatants of dendritic cells were measured by human TNF-α ELISA kit, human IL-12(p70) ELISA kit, and human TGF-β1 ELISA kit (all by BD Biosciences) and used according to the manufacturer’s instructions. DC were infected or not with BCG (MOI 5) and incubated for 3 days in the presence or not of either 3 mg/ml HyE-Ov or the different HyE-Ov purified fractions, used at the same concentrations present in the original total extract. The supernatants were collected at 24, 48 and 72 hours after stimulation and stored at -20°C until analysis.

### Statistical analysis

Statistical analysis of data was performed using Student’s t test. *p* values lower than 0.05 were considered statistically significant.

## Results

### HyE-Ov promotes antimicrobial response leading to the reduction of intracellular mycobacterial viability

**Fig 1:**
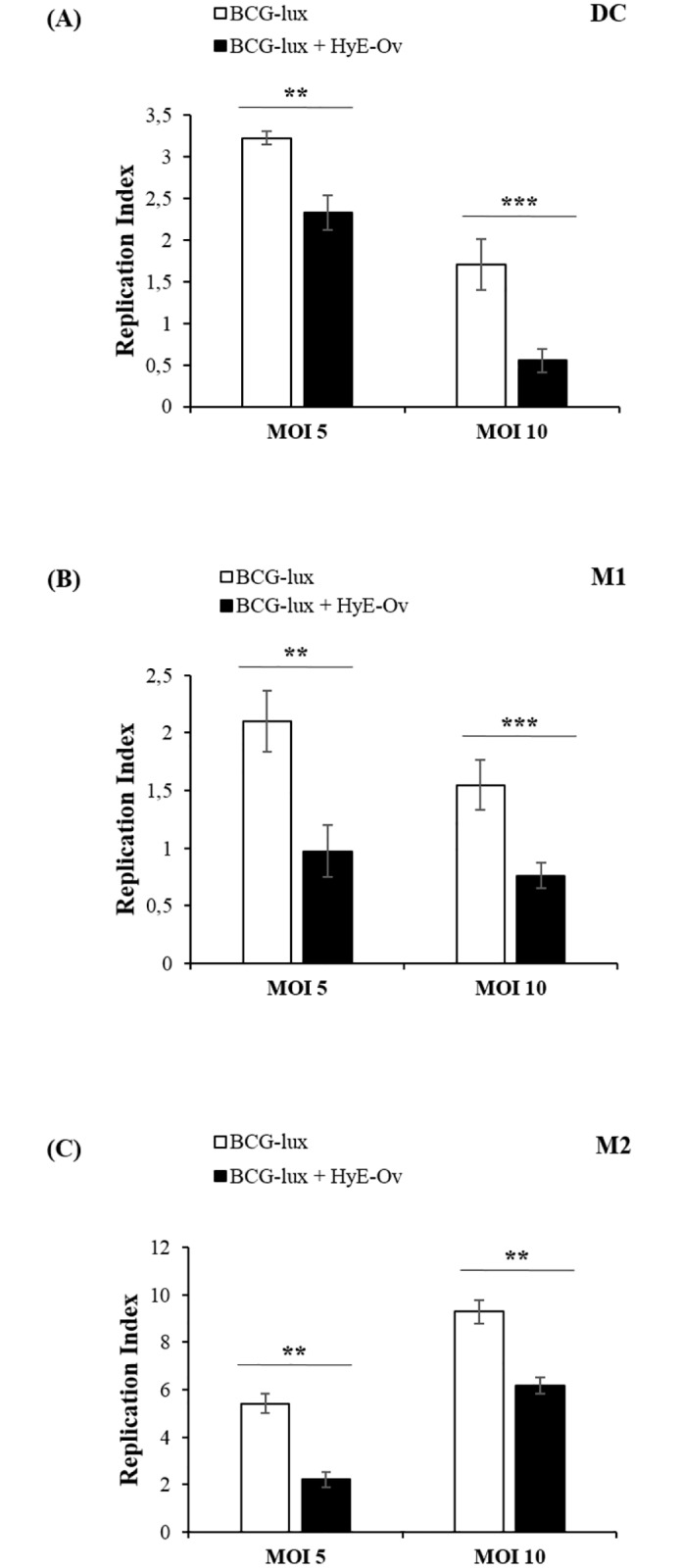
HyE-Ov promotes intracellular mycobacterial killing. DC (A), M1 (B) and M2 (C) cells (5x10^5^/well) were infected with BCG-lux for 3 hours (MOI 5 and 10) and stimulated or not with HyE-Ov (3 mg/ml of equivalent plant material). Mycobacterial growth was evaluated by luminometric analysis after 3 days (t3) from stimulation (t0). Data are shown as means ± Standard Deviation (SD) of the ratio t3/t0 of RLU values from triplicate cultures and are representative of at least 3 independent experiments performed on cells from different donors. **p<0.01 and ***p<0.0001 in comparison with non-stimulated infected cells.

In order to evaluate the capability of HyE-Ov to induce anti-mycobacterial activity, DC, M1 and M2 cells were infected with BCG-lux at the multiplicity of infection (MOI) of 5 or 10, and stimulated for 3 days with HyE-Ov. Intracellular mycobacterial viability was assessed by a luminometric analysis and the results, expressed in Fig 1, show the capability of HyE-Ov to reduce intracellular mycobacterial viability in all cell types analysed, irrespective of the MOI used. However, as a lower replication index was observed following infection with MOI 10 in DC and M1, which was associated with lower cell viability (S1 Fig), we used MOI of 5 for next *in vitro* infection experiments. Moreover, the observed reduction of intracellular mycobacterial viability following stimulation by HyE-Ov was not due to a bactericidal effect, as any direct toxicity on BCG-lux growth was observed during culture in 7H9 broth (S2 Fig). Finally, any direct cytotoxic effect was not observed on DC, M1 and M2 following stimulation with HyE-Ov at day 3 after stimulation (S3 Fig).

### HyE-Ov promotes phagosome acidification and intracellular ROS production

**Fig 2:**
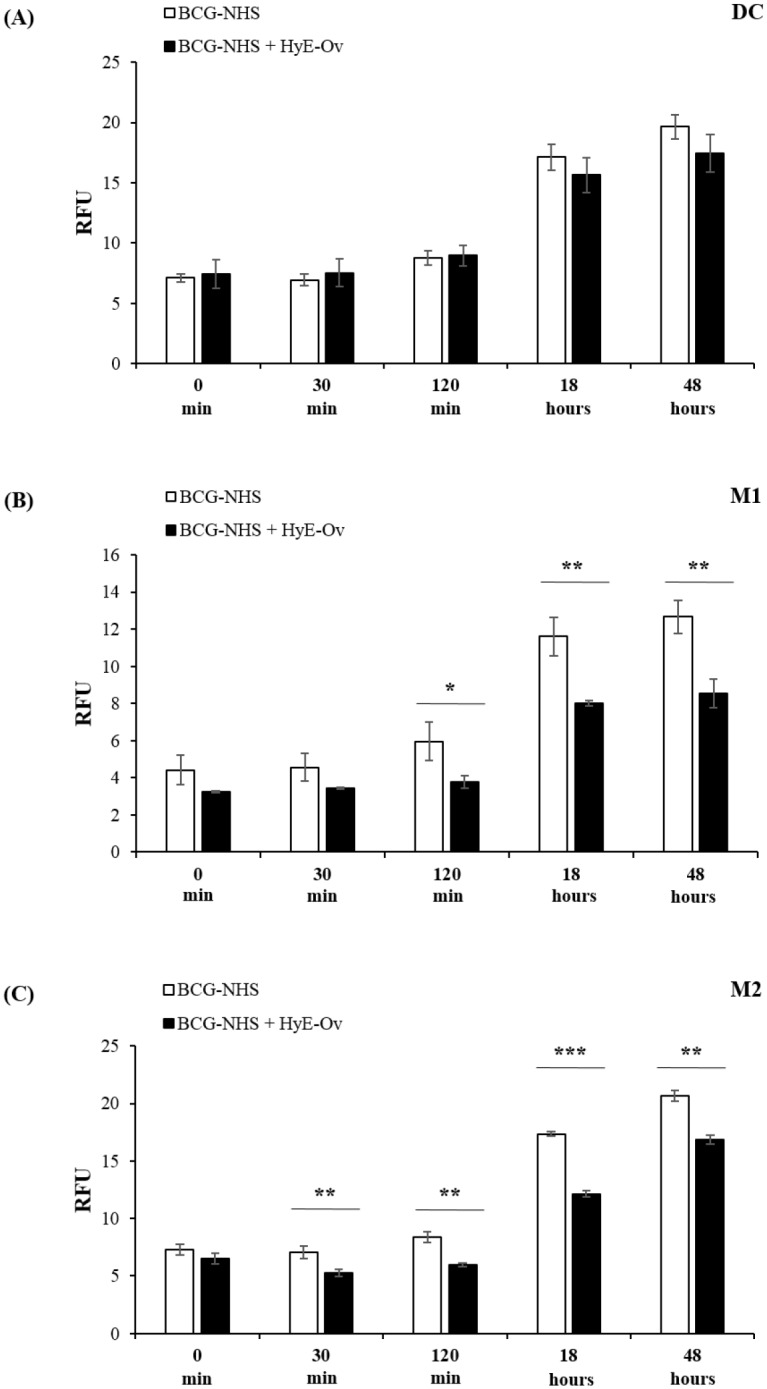
HyE-Ov promotes phagosome acidification. DC (A), M1 (B) and M2 (C) cells (10^6^/well) were infected with BCG conjugated with NHS (MOI 5) for 3 hours and then stimulated with HyE-Ov (3 mg/ml of equivalent plant material). Results are expressed as means ± SD of Relative Fluorescent Units (RFU) of triplicate culture values and are representative of at least 3 independent experiments on cells from different donors. *p<0.05, **p<0.01 and ***p<0.0001 in comparison with non-stimulated infected cells.

After phagocytosis, phagosome lumen acidification is a crucial step of phagosome maturation and many pathogens, such as *M*. *tuberculosis*, tend to hamper this process [32]. To evaluate the maturation status of the phagosomes, we monitored phagosome acidification until 48 hours post-infection, by labelling BCG with NHS, a pH sensitive fluorochrome. Fig 2B and 2C show that stimulation with HyE-Ov in M1 and M2, but not in DC (Fig 2A), significantly decreases phagosomal pH, when compared to unstimulated controls.

**Fig 3:**
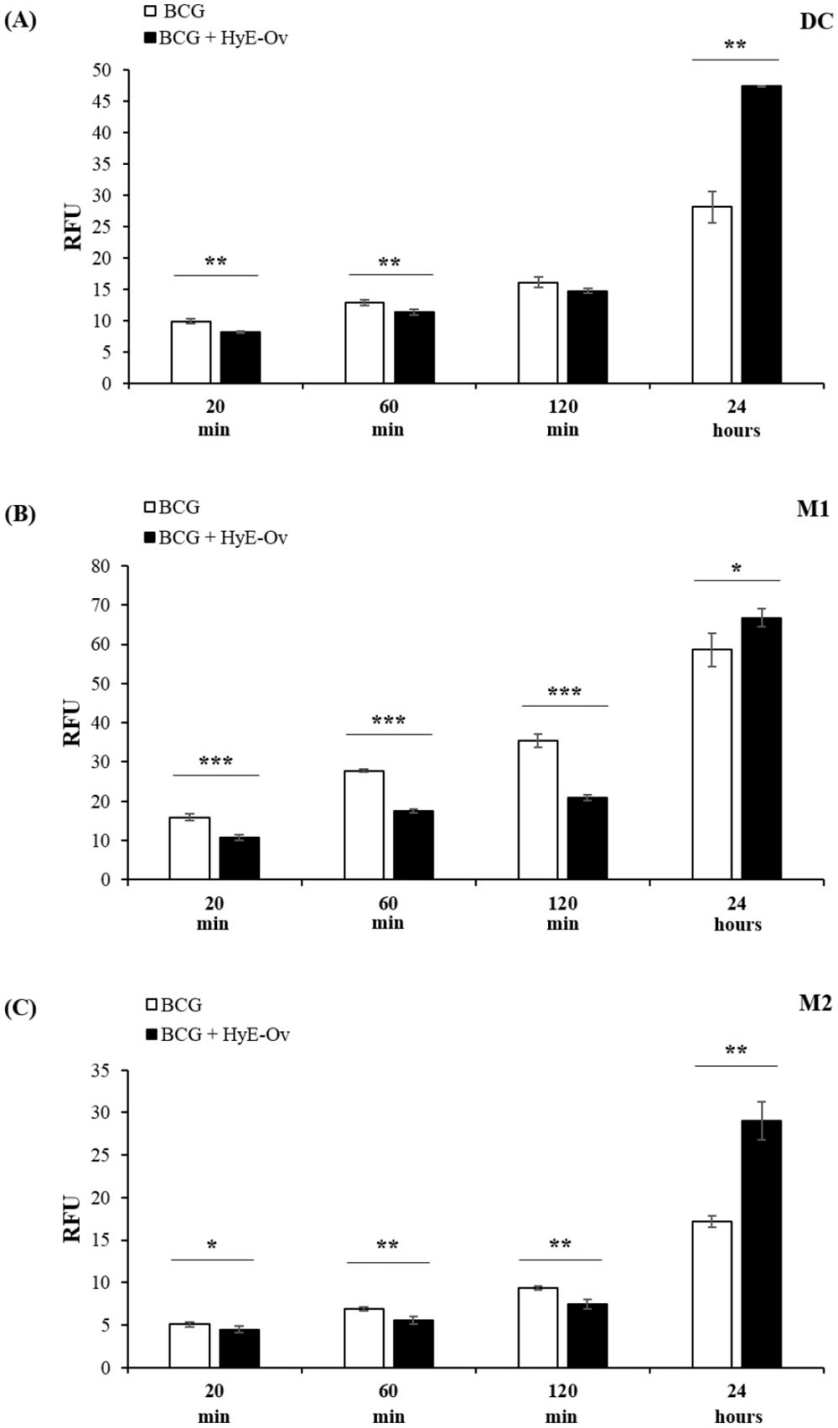
HyE-Ov promotes intracellular Reactive Oxygen Species (ROS) production. DC (A), M1 (B) and M2 (C) cells (3x10^5^/well) were infected for 3 hours with BCG (MOI 5), labelled with 10 μM of DCF and then stimulated with HyE-Ov for 20 min, 60 min, 120 min and 24 hours. Data are shown as means ± SD of RFU of culture quadruplicate values and are representative of at least 2 independent experiments performed on cells from different donors. *p<0.05, **p<0.01 and ***p<0.0001 in comparison with non-stimulated infected cells.

The production of ROS by phagosomal type 2 NADPH oxidase [33] represents an important mechanism for phagocytic cells to kill intracellular bacteria. We used DCF, a ROS sensitive fluorescent probe, to monitor the oxidative status of BCG-infected cells and to evaluate the possible role of HyE-Ov treatment in ROS production. Results, shown in Fig 3, indicate that HyE-Ov treatment of M1, M2 and DC induces an initial anti-oxidant response followed by a switch to a significant ROS generation at 24 hours after stimulation.

### HyE-Ov promotes intracellular mycobacterial killing by a pH and ROS-dependent mechanism

**Fig 4:**
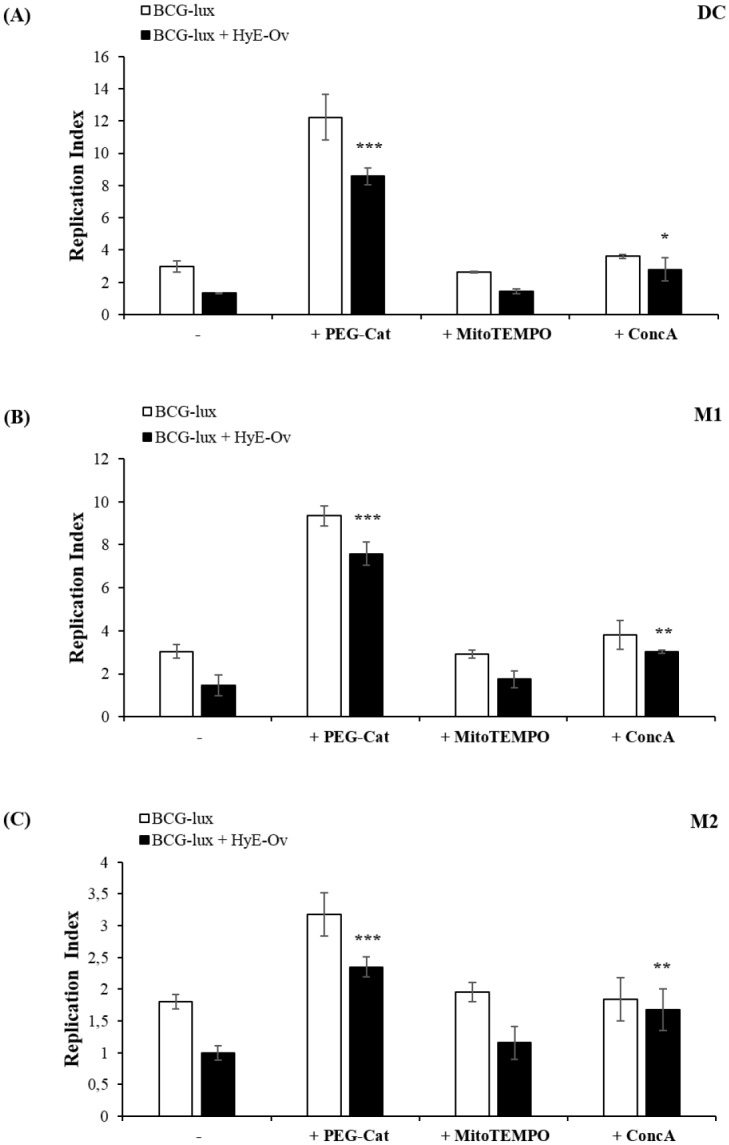
HyE-Ov promotes intracellular mycobacterial killing by a pH and ROS-dependent mechanism. DC (A), M1 (B) and M2 (C) cells (5x10^5^/well) were infected with BCG-lux for 3 hours (MOI 5) and stimulated or not with HyE-Ov (3 mg/ml of equivalent plant material) in the presence or absence of 100 U/ml PEG-Catalase, 10 μM MitoTEMPO or 10 nM Concanamycin A. Mycobacterial growth was evaluated by luminometric analysis after 3 days (t3) from stimulation (t0). Data are shown as means ± SD of the ratio t3/t0 of RLU values from triplicate cultures and are representative of at least 2 independent experiments performed on cells from different donors. *p<0.05, **p<0.01 and ***p<0.0001 in comparison with HyE-Ov stimulated infected cells.

To evaluate whether intracellular mycobacterial killing induced by HyE-Ov stimulation is mediated by phagosome acidification and ROS generation, we monitored the intracellular mycobacterial viability in the presence of inhibitors of phagosome acidification and of ROS generation. In details, we used Concanamycin A (ConcA), a specific inhibitor of V-ATPases, which blocks phagosome acidification, and polyethylene glycol-Catalase (PEG-Cat), which converts hydrogen peroxide to water and oxygen, thus reducing ROS. In addition, we used MitoTEMPO, a specific scavenger of mitochondrial superoxide, in order to evaluate the involvement of mitochondrial ROS. Results, shown in Fig 4, indicate that intracellular mycobacterial growth is completely restored in HyE-Ov stimulated cells after exposure to ConcA or PEG-Cat. Conversely, the stimulation with MitoTEMPO does not affect intracellular mycobacterial viability when compared with HyE-Ov treated cells, only.

### HyE-Ov down-regulates inflammatory response by reducing TNF-α and IL-12 release and increasing TGF-β production

**Fig 5:**
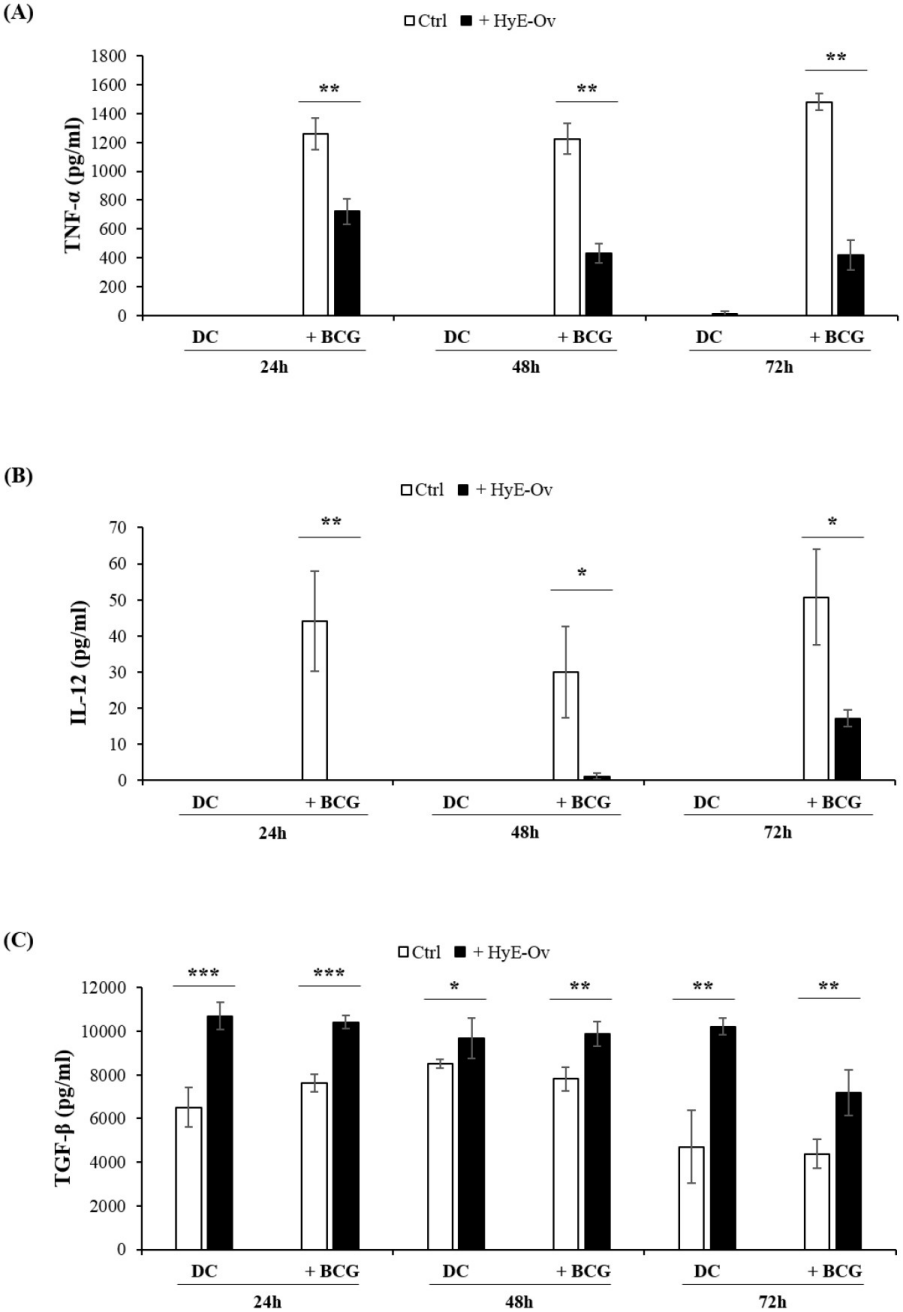
HyE-Ov down-regulates inflammatory response by reducing TNF-α (A) and IL-12 (B) release and increasing TGF-β release (C). DC (10^6^/well) were infected or not with BCG (MOI 5) for 3 hours and were stimulated or not with HyE-Ov. At 24, 48 and 72 hours after stimulation, supernatants were collected, stored at -20°C and then analysed by ELISA. Data are expressed as means ± SD of culture triplicate values and are representative of at least 2 independent experiments performed on cells from different donors. *p<0.05, **p<0.01 and ***p<0.0001 in comparison with unstimulated either uninfected or BCG-infected control.

*O*. *vulgare* was shown to possess anti-inflammatory properties [17, 19], but the relevance of this mechanism during infections are not fully investigated. We hypothesized that the interference with the secretion of cytokines, known to modulate inflammation, could represent a mechanism of action lead by HyE-Ov during *in vitro* infection with BCG. We therefore measured the levels of TNF-α, IL-12 and TGF-β in the supernatants of DC infected or not with BCG at the MOI of 5, and treated or not with HyE-Ov. The levels of cytokines were then analysed at 24, 48 and 72 hours from HyE-Ov stimulation. Results indicate that HyE-Ov treatment significantly reduces the levels of TNF-α (Fig 5A) and IL-12 (Fig 5B) in BCG-infected cells whereas enhances the production of TGF-β (Fig 5C) in both BCG-infected and uninfected cells.

### Evaluation of the different fractions from HyE-Ov on antimicrobial and inflammatory response

**Fig 6:**
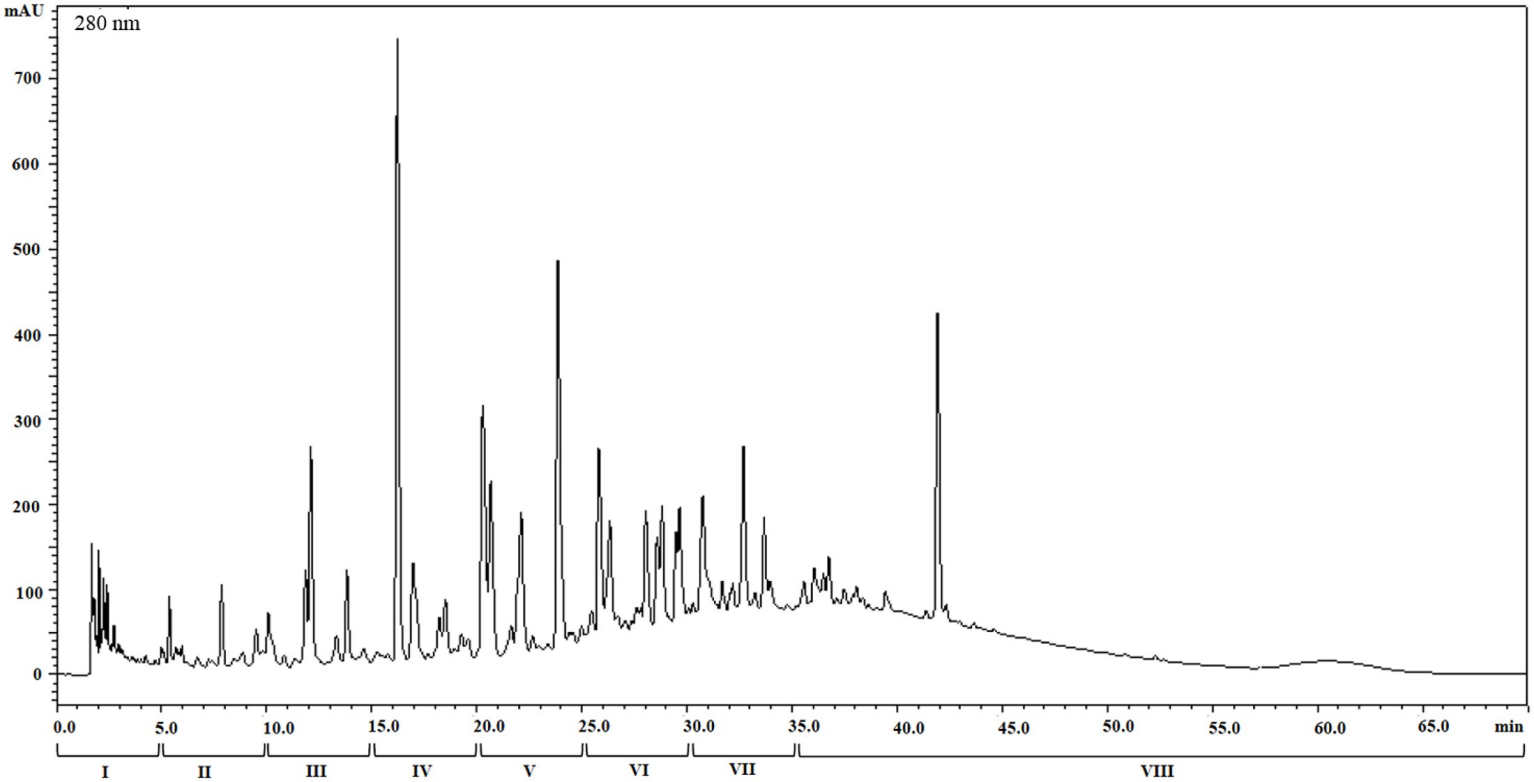
Chromatogram of HyE-Ov. Representative UV-visible absorption chromatographic profile of HyE-Ov obtained by HPLC-DAD analysis at 280 nm (absorption wavelength of phenolic acids) was reported.

**Fig 7:**
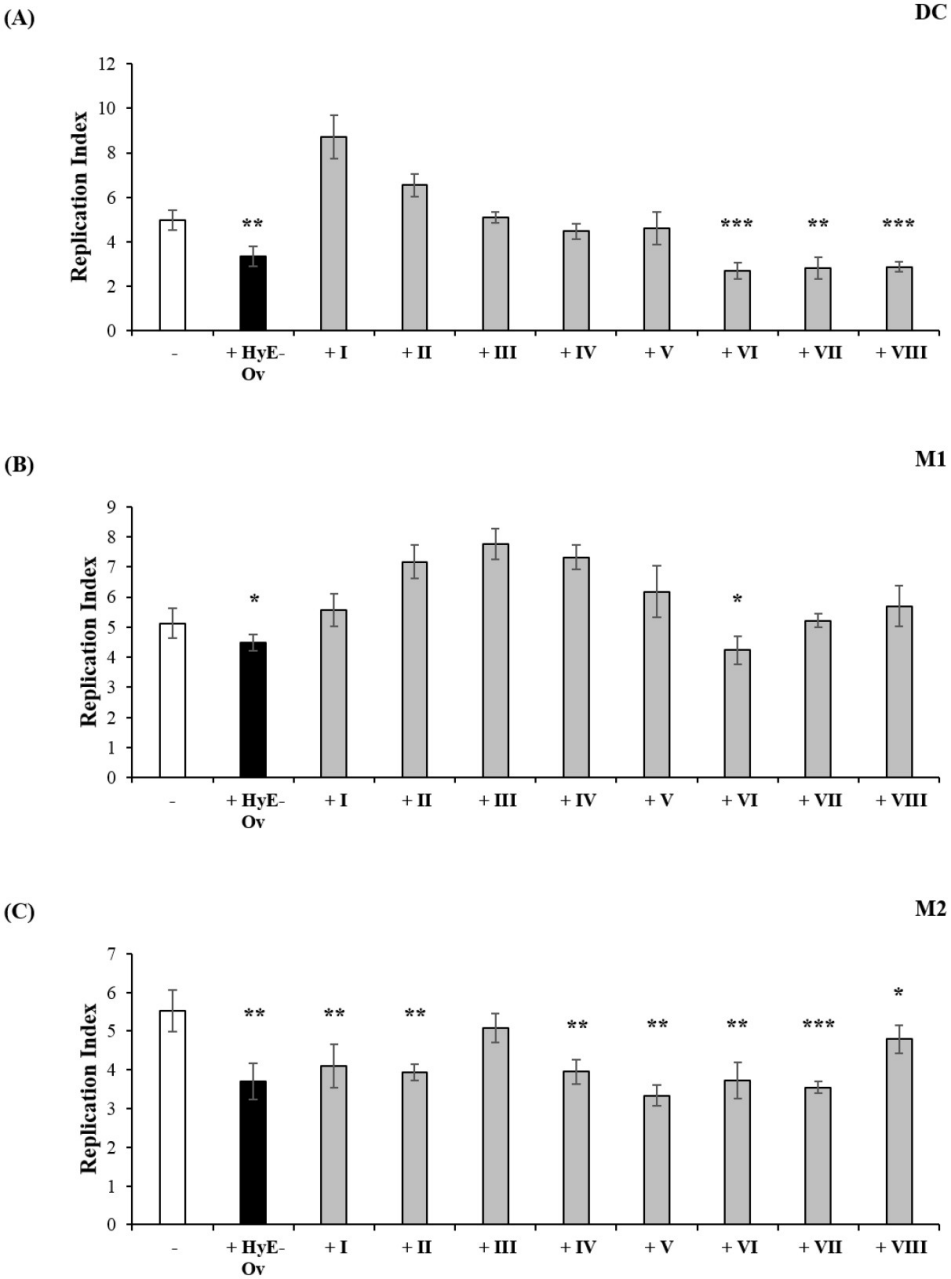
Effects of fractions from HyE-Ov on antimicrobial innate immune response. DC (A), M1 (B) and M2 (C) cells (5x10^5^/well) were infected with BCG-lux for 3 hours (MOI 5) and stimulated or not with HyE-Ov or with eight HyE-Ov derived fractions, used at the same concentrations present in the total extract. Mycobacterial growth was evaluated by luminometric analysis after 3 days from stimulation (t0). Data are shown as means ± SD of the ratio t3/t0 of RLU values from triplicate cultures and are representative of at least 2 independent experiments performed on cells from different donors. *p<0.05, **p<0.01 and ***p<0.0001 in comparison with non-stimulated infected cells.

Phytocomplex represents the set of all secondary metabolites extracted from the tissues of a specific plant organism, including the active molecule/molecules (e.g. sugars, aminoacids) responsible for its documented biological effects. In order to identify the possible presence of specific metabolite/s responsible for the observed biological effects, HyE-Ov was separated into eight fractions, by HPLC-DAD (Fig 6). Thereafter, the biological activity of all different fractions was tested on the different cell types infected with BCG-lux and luminescence, indicative of mycobacterial viability, was evaluated at day 3 after infection. Results indicate that fractions VI, VII, VIII were active on DC (Fig 7A), fraction VI only showed effects on M1 (Fig 7B) and almost all fractions, except fraction III, were bioactive on M2 cells (Fig 7C).

**Fig 8:**
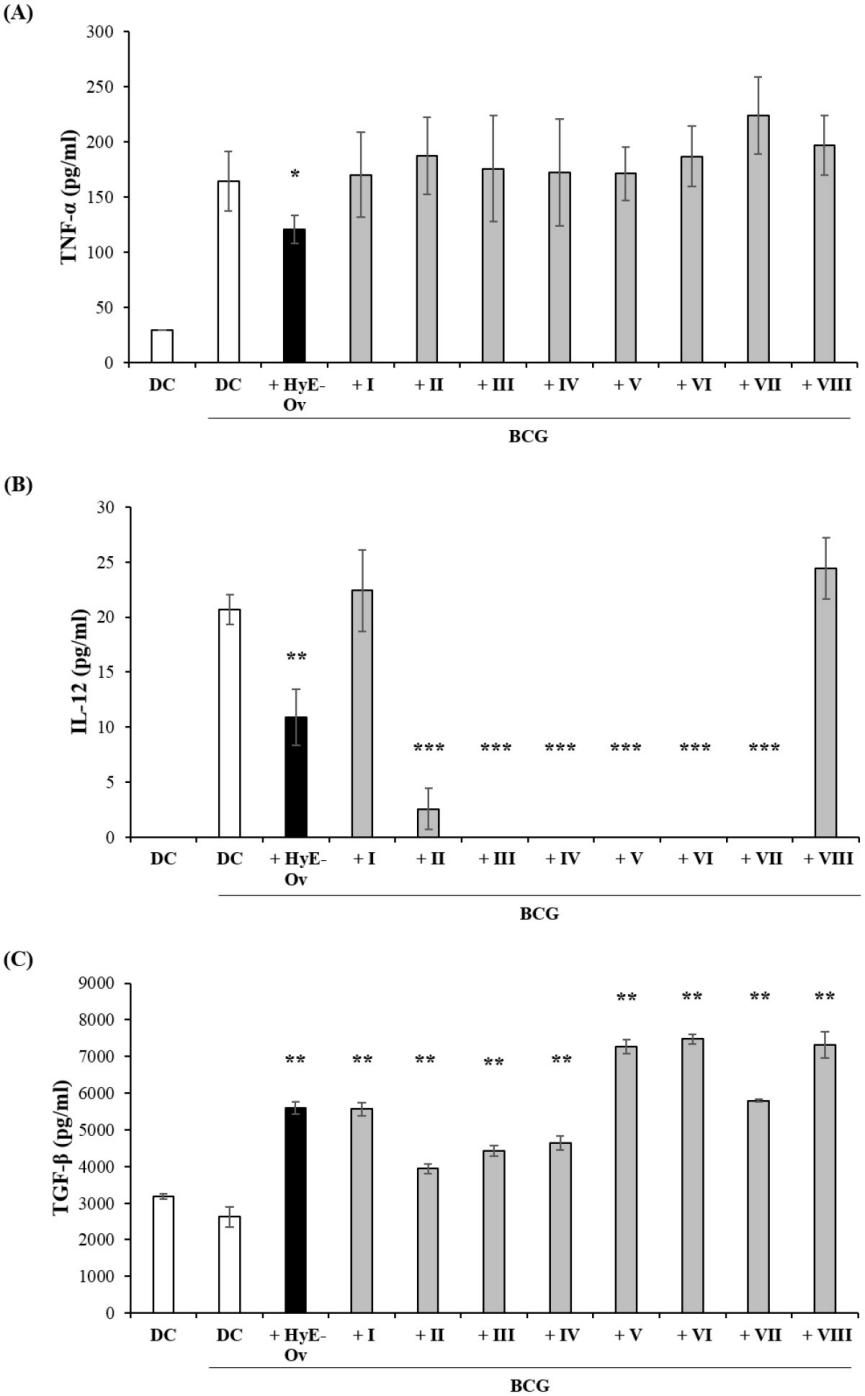
Effects of fractions from HyE-Ov on inflammatory response. DC (10^6^/well) were infected with BCG (MOI 5) for 3 hours and then stimulated or not with HyE-Ov or with eight HyE-Ov derived fractions. At 72 hours after stimulation, supernatants were collected, stored at -20°C and then analysed by ELISA. Data are expressed as means ± SD of culture triplicate and are representative of 2 independent experiments performed on cells from different donors. *p<0.05, **p<0.01 and ***p<0.0001 in comparison with non-stimulated infected cells.

The analysis of inflammatory/anti-inflammatory response performed on BCG-infected DC following stimulation with the 8 fractions shows that any fraction was not able to reproduce the observed HyE-Ov induced inhibition of TNF-α (Fig 8A), all fractions except fraction I and VIII inhibit the BCG induced production of IL-12 (Fig 8B), and all fractions reproduce enhancement of TGF-β (Fig 8C).

## Discussion

All biomolecules, either of natural or of synthetic origin, able to suppress or stimulate the immune system, are known as immunomodulators and the interest for their identification among plants is increased during the last years [34]. In fact, plants or their extracts may represent a source of products with immunomodulatory activity and, as such, can be used as nutraceuticals, substances that have a beneficial effect on health, or in phytotherapy, an ancient practice consisting of use of plants for the prevention or treatment of diseases. A number of studies have described significant anti-inflammatory activity and direct bactericidal properties of *O*. *vulgare* derived essential oils [7], but issues concerning tolerability and toxicities limited its uses in human subjects [35]. Water soluble, hydroalcoholic and methanolic extracts of the plant, which are normally characterized by good tolerability, have also been reported to show anti-inflammatory, anti-oxidant and direct bactericidal features [16, 17]. However, studies on the identification of plant extracts capable to promote antimicrobial activity in innate immune cells by simultaneously limiting potentially tissue-damaging inflammatory response are still lacking. In the present study, we showed the capability of HyE-Ov to enhance anti-microbial response of innate immune cells, such as dendritic cells, type-1 and type-2 macrophages in a model of *in vitro* BCG infection. These cell types have been used as they are the preferential targets of *Mycobacterium tuberculosis* [36] and a correct balance in the response of these different cell types in the granuloma is necessary both to control the infection and to limit the tissue damage [37]. Despite of differences in intracellular mycobacterial replication among the different cell types [38, 39], HyE-Ov stimulation leads to a significant reduction of intracellular mycobacterial growth in all cell types, independently by the MOI used and by the basal anti-mycobacterial response.

After phagocytosis, the resulting phagosome is subjected to a series of fission and fusion events with early and late endosomes, and ultimately lysosomes, causing its maturation to phagolysosome, the final organelle deputed to the full microbicidal action [40]. During this process, phagosomes acquire vacuolar ATPase (vATPase), leading to the progressive intravacuolar acidification, and type 2 NADPH oxidase, a phagosome associated enzyme generating large quantities of reactive oxygen species, which together with the acquisition of proteases, nucleases, and lipases contribute to the generation of a full microbicidal environment of phagolysosomes [41]. Progressive acidification of maturing phagosomes is crucial for antigen degradation and to make available protons as major ionic species contributing to NADPH oxidase charge compensation during phagosomal ROS production. This causes an intimate and complex relationship between phagosomal pH and ROS generation [42]. Results reported herein show that anti-mycobacterial activity induced by HyE-Ov is pH dependent in all cell types tested. In agreement with previously reported anti-oxidant activity of alcoholic extract of *O*. *vulgare* [13], results reported herein also show an initial reduction of ROS generation in the three cell types analysed that, however, turns to an oxidative response activation, detectable at the late stimulation time (24 hours) in all BCG-infected cell types. Moreover, experiments using catalase, which converts hydrogen peroxide to water and oxygen and thus reduces ROS activity, and mitoTEMPO, a scavenger of mitochondrial ROS, support the phagosomal origin of bactericidal ROS induced by HyE-Ov, although mitochondrial ROS have been reported to contribute to antimicrobial activity of phagocytes [43]. Thus, our results support the hypothesis that HyE-Ov induced ROS contribute to the intracellular mycobacterial killing in a late phase of phagocytosis, when NOX-2 is assembled and activated on mature phagosomes [44]. The apparent discrepancy of DC, which fail to further acidify their phagosome following HyE-Ov stimulation although they were sensitive to HyE-Ov treatment in terms of intracellular mycobacteria killing, may be explained by the limited propensity of these cells to phagosome acidification, in comparison with macrophages [45]. The limited phagosomal acidification may represent a DC strategy to ensure enhanced antigen bioavailability and antigen presentation efficiency [46]. In this context, the phagosome acidification occurring in DC, although partial and limited, nevertheless supports NOX-2 activity, which can be compromised in the absence of the compensating electrogenic activity provided by phagosomal vATPase [47].

In immunopathology, tissue damage is caused by excessive inflammatory response of immune system. In patients with tuberculosis, chronic inflammation leads to initial granuloma formation followed by alterations of the normal architecture and function of lung tissue during active stages of disease [48, 49]. The main soluble mediator of the granulomatous inflammatory response is TNF-α, which is mainly produced by macrophages and dendritic cells. In this context, an ideal drug aimed at strengthening the host’s antimicrobial defences should increase innate cell capability to kill mycobacteria, but also limit the inflammatory response responsible for tissue damage [50,51]. Together, data reported herein demonstrate that HyE-Ov stimulation of DC significantly reduces the BCG-induced TNF-α and IL-12 production by simultaneously increasing TGF-β response, in agreement with previous reports obtained on innate and adaptive immune cells [17, 19] where different *O*. *vulgare* extracts were used. In addition, our data show that treatment of DC with HyE-Ov also increases antimicrobial response suggesting that HyE-Ov is endowed with the potential to simultaneously kill bacteria and reduce inflammation, fulfilling, at least *in vitro*, the requirements of an ideal treatment for mycobacterial infections.

The isolation from plants of novel compounds with therapeutic value is an important strategy in the drug discovery, which has led in the past to the identification of many currently used drugs [34]. In order to identify the compounds present within the oregano extract, which may be responsible for the observed biological effect, preliminary fractionation of the extract has been carried out by HPLC and the single fractions tested separately in terms of pro-mycobactericidal and anti-inflammatory activity. Results reported herein indicate that several fractions may activate a mycobactericidal response independently by the cell type, whereas others show a more specific cell target requirement. These results suggest that the single fractions may contain different compounds capable to induce antimicrobial response in the context of one cell type (e.g. fraction VI, VII, and VIII in DC or fractions I, II, IV, V, VI, VII and VIII in M2). Moreover, the evidence that one single fraction may be active on one cell type and not on another may reflect differences in the molecular requirements for the formation of a fully microbicidal phagolysosome compartment in M1, M2 and DC, as reported [45, 52]. A similar analysis was performed in order to identify possible compounds from oregano extract responsible for the observed anti-inflammatory activity. Results reported herein show that i) all fractions were able to increase level of TGF-β, ii) fractions from II to VII reduced IL-12 production, and iii) any fraction was not active in terms of TNF-α inhibition, on BCG infected DC. These results support the notion that more than one compound can be active on TGF-β and IL-12 production and that the observed effects on TNF-α can be mediated by a phytocomplex, namely by a collection of plant secondary metabolites and primary molecules, which may operate in combination and so justify the bioactivity of the whole plant extract. In fact, it is known that phytocomplex may have properties that the single molecules present in it do not show. In this context, it has been demonstrated that lycopene phytocomplex, but not pure lycopene, was bioactive on HL60 human leukemia cells [53].

In conclusion, results reported in the present study show that hydroalcoholic extract of *O*. *vulgare* contain a plethora of metabolites with potential immunotherapeutic value to enhance innate anti-microbial activity by simultaneously limiting inflammatory response. On these grounds, HyE-Ov as a whole may represent a possible adjuvant immunoprophylactic or immunotherapeutic tool for the control of recurrent bacterial infections, especially those responsible of immunopathologic reactions, occurring in patients with chronic diseases and which are at risk of developing antibiotic-resistance.

## Supporting information

S1 Fig Host cell viability after infection with BCG-lux at MOI 5 and MOI 10. DC (A), M1 (B) and M2 (C) (2x10^5^/well) were infected with BCG-lux (MOI 5 and 10) for 3 hours, were incubated for 3 days at 37°C with 5% CO_2_ and were subjected to MTT assay. Data are expressed as means ± SD of % of cell viability of triplicate cultures and are representative of 2 independent experiments performed on cells from different donor. *p<0.05 and ***p<0.0001 in comparison with uninfected cells.
(TIF)

S2 Fig Hydroalcoholic extract of *O*. *vulgare* (HyE-Ov) does not exert any direct toxic effects on mycobacteria. BCG-lux was cultured in 7H9 medium in the presence or absence of the extract and the mycobacterial growth was monitored for 2 weeks by a luminometric assay. Data are expressed as Replication Index, calculated as the ratio between the Relative Luminescence Units (RLU) obtained at the day indicated in figure and the RLU value obtained at the beginning of culture (t0). Data are representative of 2 independent cultures.
(TIF)

S3 Fig *O*. *vulgare* extract does not exert any direct toxic effects on host cells. DC (A, B), M1 (C, D) and M2 (E, F) (2x10^5^/well) were stimulated with HyE-Ov at different concentration (1, 3, 9 and 27 mg/ml of equivalent plant material) for 3 days. Cells and supernatants were subjected to MTT Assay (A, C, E) and to CytoTox 96 Assay (B, D, F). Data are expressed as means ± SD of % of cell viability or % of cytotoxicity of triplicate cultures and are representative of 2 independent experiments performed on cells from different donors. *p<0.05 and **p<0.01 in comparison with non-stimulated cells.
(TIF)

S1 Method MTT assay. In order to evaluate the cell viability after infection, all cell types were infected with BCG-lux (MOI 5 and 10) for 3 hours. Washings were performed to block infection and to remove eventual extracellular bacteria, and cells were incubated for 3 days at 37°C with 5% CO_2_. MTT assay (Molecular Probe) was performed according to the manufacturer’s instructions. The MTT assay is based on the cleavage of the yellow tetrazolium salt MTT (3-(4,5-Dimethylthiazol-2-yl)-2,5-diphenyltetrazolium bromide) to purple formazan crystal in metabolically active cells. The formazan is then solubilized, and the concentration determined by optical density at 540 nm. The assay is sensitive with the colorimetric signal proportional to the viable cell number. As negative control, all cell types were treated with 0.1% saponin at 37°C for 30 min. Data are shown as means ± SD of % of cell viability of triplicate cultures. % Cell viability = 100 x Experimental OD_540nm_ / Positive Control OD_540nm_. Data are representative of 2 independent experiments performed on cells from different donors.
(DOCX)

S2 Method Assessment of direct mycobactericidal effect of HyE-Ov. BCG-lux was stored at -80°C and, after thawing, centrifuged and suspended in Middlebrook 7H9 (Difco) broth supplemented with 10% ADC (albumin, dextrose and catalase), 0.05% Tween 80 and 50 μg/ml Hygromycin B (Invitrogen). Bacteria were sonicated, in a bath sonicator, for 3 min to remove mycobacterial clumps and dispensed equally into two flasks, in the presence or absence of HyE-Ov (3 mg/ml of equivalent plant material). Mycobacterial growth was monitored for 2 weeks by a luminometric assay, as described [28]. Briefly, luminometric analysis was performed using PBS, mycobacterial suspension and 1% decanal (as luciferase substrate), at the ratio 8:1:1. At these experimental conditions, mycobacterial viability is directly proportional to the intensity of luminescence [30].
(DOCX)

S3 Method Cytotoxicity assays. In order to analyse the possible cytotoxic effect of HyE-Ov on dendritic cells (DC), type-1 macrophages (M1) and type-2 macrophages (M2), all cell types were stimulated with HyE-Ov at different concentrations (1, 3, 9 and 27 mg/ml of equivalent plant material). After 3 days of stimulations, cell viability was assessed by MTT assay (Molecular Probe), used according to the manufacturer’s instructions. Cells treated with 0.1% saponin at 37°C for 30 min and unstimulated cells served as a negative and positive control, respectively. Data are shown as means ± SD of % of cell viability of triplicate cultures. % Cell viability = 100 x Experimental OD_540nm_ / Positive Control OD_540nm._ Data are representative of 2 independent experiments performed on cells from different donors. Simultaneously, supernatants were collected and tested by CytoTox 96 Assay (Promega) according to the manufacturer’s instructions. In particular, the CytoTox 96 Assay measures lactate dehydrogenase (LDH), a stable cytosolic enzyme that is released upon cell lysis, by means of the conversion of a tetrazolium salt (iodonitrotetrazolium violet; INT) into a red formazan product. The amount of colour formed is determined by optical density at 490 nm and is proportional to the number of lysed cells. Cells treated with 0.1% saponin at 37°C for 30 min and unstimulated cells served as a positive and negative control, respectively. Data are shown as means ± SD of % of cytotoxicity of triplicate cultures. % Cytotoxicity = 100 x Experimental OD_490nm_ / Positive Control OD_490nm._ Data are representative of 2 independent experiments performed on cells from different donors.
(DOCX)

## References

[1] Thomford NE, Senthebane DA, Rowe A, Munro D, Seele P, Maroyi A, et al Natural Products for Drug Discovery in the 21st Century: Innovations for Novel Drug Discovery. Int J Mol Sci. 2018; 19: 10.3390/ijms19061578 29799486 PMC6032166

[2] Patridge E, Gareiss P, Kinch MS, Hoyer D. An analysis of FDA-approved drugs: natural products and their derivatives. Drug Discov Today. 2016; 21:204–207. 10.1016/j.drudis.2015.01.009 25617672

[3] Butler MS, Robertson AA, Cooper MA. Natural product and natural product derived drugs in clinical trials. Nat Prod Rep. 2014; 31:1612–1661. 10.1039/c4np00064a 25204227

[4] Gupta VK, Kumar MM, Bisht D. and Kaushik A. Plants in our combating strategies against Mycobacterium tuberculosis: progress made and obstacles met. Pharm Biol. 2017; 55:1536–1544. 10.1080/13880209.2017.1309440 28385088 PMC6130758

[5] Castro M, Preto M, Vasconcelos V, Urbatzka R. Obesity: The Metabolic Disease, Advances on Drug Discovery and Natural Product Research. Curr Top Med Chem. 2016; 16:2577–2604. 10.2174/1568026616666160415155644 27086785

[6] Şahin F, Güllüce M, Daferera D, Sökmen A, Sökmen M, Polissiou M, et al Biological activities of the essential oils and methanol extract of Origanum vulgare ssp. vulgare in the Eastern Anatolia region of Turkey. Food control. 2004; 15:549–557. 10.1016/j.foodcont.2003.08.009

[7] Pezzani R, Vitalini S, Iriti M. Bioactivities of *Origanum vulgare* L.: an update. Phytochem Rev. 2017; 16:1253–1268. 10.1007/s11101-017-9535-z

[8] Leyva-López N, Gutiérrez-Grijalva EP, Vazquez-Olivo G, Heredia JB. Essential Oils of Oregano: Biological Activity beyond Their Antimicrobial Properties. Molecules. 2017; 22:E989 10.3390/molecules22060989 28613267 PMC6152729

[9] Zhang XL, Guo YS, Wang CH, Li GQ, Xu JJ, Chung HY, et al Phenolic compounds from *Origanum vulgare* and their antioxidant and antiviral activities. Food Chem. 2014; 152:300–306. 10.1016/j.foodchem.2013.11.153 24444941

[10] Ivanova D, Gerova D, Chervenkov T. and Yankova T. Polyphenols and antioxidant capacity of Bulgarian medicinal plants. J Ethnopharmacol. 2005; 96:145–150. 10.1016/j.jep.2004.08.033 15588663

[11] Tusevski O, Kostovska A, Iloska A, Trajkovska L, Simic SG. Phenolic production and antioxidant properties of some Macedonian medicinal plants. Centr Eur J Biol. 2014; 9:888–900. 10.2478/s11535-014-0322-1

[12] Skendi A, Irakli M, Chatzopoulou P. Analysis of phenolic compounds in Greek plants of Lamiaceae family by HPLC. J Appl Res Med Aromat Plants. 2017; 6:62–69. 10.1016/j.jarmap.2017.02.001

[13] Coccimiglio J, Alipour M, Jiang ZH, Gottardo C and Suntres Z. Antioxidant, antibacterial, and cytotoxic activities of the ethanolic *Origanum vulgare* extract and its major constituents. Oxid Med Cell Longev. 2016; 2016:1404505 10.1155/2016/1404505 27051475 PMC4804097

[14] Teixeira B, Marques A, Ramos C, Serrano C, Matos O, Neng NR and et al Chemical composition and bioactivity of different oregano (*Origanum vulgare*) extracts and essential oil. J Sci Food Agric. 2013; 93:2707–2714. 10.1002/jsfa.6089 23553824

[15] Ličina BZ, Stefanović OD, Vasić SM, Radojević ID, Dekić MS, Čomić LR. Biological activities of the extracts from wild growing *Origanum vulgare* L. Food Control. 2013; 33:498–504. 10.1016/j.foodcont.2013.03.020

[16] Martins N, Barros L, Santos-Buelga C, Henriques M, Silva S and Ferreira ICFR. Decotion, infusion and hydroalcoholic extract of *Origanum vulgare* L.: different performances regarding bioactivity and phenolic compounds. Food Chem. 2014; 158:73–80. 10.1016/j.foodchem.2014.02.099 24731316

[17] Vujicic M, Nikolic I, Kontogianni VG, Saksida T, Charisiadis P, Orescanin-Dusic Z et al Methanolic extract of *Origanum vulgare* ameliorates type 1 diabetes through antioxidant, anti-inflammatory and anti-apoptotic activity. Br J Nutr. 2015;113:770–782. 10.1017/S0007114514004048 25671817

[18] Gunawardena D, Shanmugam K, Low M, Bennett L, Govindaraghavan S, Head R and et al Determination of anti-inflammatory activities of standardised preparations of plant- and mushroom-based foods. Eur J Nutr. 2014;53:335–343. 10.1007/s00394-013-0531-9 23653285

[19] Ocaña-Fuentes A, Arranz-Gutiérrez E, Señorans FJ and Reglero G. Supercritical fluid extraction of oregano (*Origanum vulgare*) essentials oils: Anti-inflammatory properties based on cytokine response on THP-1 macrophages. Food Chem Toxicol. 2010;48:1568–1575. 10.1016/j.fct.2010.03.026 20332013

[20] Bukovská A, Čikoš Š, Juhás Š, Il’kova G, Rehák P and Koppel J. Effects of a Combination of Thyme and Oregano Essential Oils on TNBS-Induced Colitis in Mice. Mediators Inflamm. 2007; 2007:23296 10.1155/2007/23296 18288268 PMC2233768

[21] Liu Q, Meng X, Li Y, Zhao CN, Tang GY, Li HB. Antibacterial and Antifungal Activities of Spices. Int J Mol Sci. 2017;18: E1283 10.3390/ijms18061283 28621716 PMC5486105

[22] Nardoni S, Giovanelli S, Pistelli L, Mugnaini L, Profili G, Pisseri F et al In Vitro Activity of Twenty Commercially Available, Plant-Derived Essential Oils against Selected Dermatophyte Species. Nat Prod Commun. 2015; 10:1473–1478. 26434145

[23] Maida I, Lo Nostro A, Pesavento G, Barnabei M, Calonico C, Perrin E and et al Exploring the Anti-Burkholderia cepacia Complex Activity of Essential Oils: A Preliminary Analysis. Evid Based Complement Alternat Med. 2014; 2014:573518 10.1155/2014/573518 24701243 PMC3950482

[24] Gutiérrez-Grijalva EP, Picos-Salas MA, Leyva-López N, Criollo-Mendoza MS, Vazquez-Olivo G, Heredia JB. Flavonoids and Phenolic Acids from Oregano: Occurrence, Biological Activity and Health Benefits. Plants (Basel). 2017; 7:E2 10.3390/plants7010002 29278371 PMC5874591

[25] World Health Organization. Global tuberculosis report 2018. http://www.who.int/tb/publications/global_report/en/

[26] Zumla A, Rao M, Wallis RS, Kaufmann SH, Rustomjee R, Mwaba P et al Host-directed therapies for infectious diseases: current status, recent progress, and future prospects. Lancet Infect Dis. 2016; 16:e47–63. 10.1016/S1473-3099(16)00078-5 27036359 PMC7164794

[27] Worthington RJ, Melander C. Combination approaches to combat multidrug-resistant bacteria. Trends Biotechnol. 2013;31: 177–84. 10.1016/j.tibtech.2012.12.006 23333434 PMC3594660

[28] Poerio N, Bugli F, Taus F, Santucci MB, Rodolfo C, Cecconi F et al Liposomes loaded with bioactive lipids enhance antibacterial innate immunity irrespective of drug resistance. Sci Rep. 2017;7:45120 10.1038/srep45120 28345623 PMC5366871

[29] Taus F, Santucci MB, Greco E, Morandi M, Palucci I, Mariotti S et al Monosodium Urate Crystals Promote Innate Anti-Mycobacterial Immunity and Improve BCG Efficacy as a Vaccine against Tuberculosis. PLoS ONE 2015; 10:e0127279 10.1371/journal.pone.0127279 26023779 PMC4449037

[30] Kampmann B, Tena GN, Mzazi S, Eley B, Young DB and Levin M. Novel human in vitro system for evaluating antimycobacterial vaccines. Infect Immun. 2004; 72:6401–6407. 10.1128/IAI.72.11.6401-6407.2004 15501770 PMC522995

[31] Pethe K, Swenson DL, Alonso S, Anderson J, Wang C, Russell DG. Isolation of *Mycobacterium tuberculosis* mutants defective in the arrest of phagosome maturation. Proc Natl Acad Sci USA. 2004; 101:13642–13647. 10.1073/pnas.0401657101 15340136 PMC518761

[32] Dey B. and Bishai WR. Crosstalk between *Mycobacterium tuberculosis* and the host cell. Semin Immunol. 2014; 26: 486–469. 10.1016/j.smim.2014.09.002 25303934 PMC4250340

[33] Babior BM. NADPH oxidase. Curr Opin Immunol. 2004; 16:42–47. 10.1016/j.coi.2003.12.001 14734109

[34] Jantan I, Ahmad W. and Bukhari SNA. Plant-derived immunomodulators: an insight on their preclinical evaluation and clinical trials. Front Plant Sci. 2015; 6:655 10.3389/fpls.2015.00655 26379683 PMC4548092

[35] Bakkali F, Averbeck S, Averbeck D, Idaomar M. Biological effects of essential oils--a review. Food Chem Toxicol. 2008; 46: 446–475. 10.1016/j.fct.2007.09.106 17996351

[36] Liu CH., Liu H. and Ge B. Innate immunity in tuberculosis: host defense vs pathogen evasion. Cell Mol Immunol. 2017; 14:963–975. 10.1038/cmi.2017.88 28890547 PMC5719146

[37] Flynn JL. Mutual attraction: does it benefit the host or the bug? Nat Immunol. 2004; 5:778–779. 10.1038/ni0804-778 15282559

[38] Verreck FAW., de Boer T., Langenberg DML., Hoeve MA., Kramer M., Vaisberg E, et al Human IL-23-producing type 1 macrophages promote but IL-10-producing type 2 macrophages subvert immunity to (myco)bacteria. Proc Natl Acad Sci USA. 2004; 101: 4560–4565. 10.1073/pnas.0400983101 15070757 PMC384786

[39] Tailleux L, Neyrolles O., Honoré-Bouakline S., Perret E., Sanchez F., Abastado JP. et al Constrained intracellular survival of *Mycobacterium tuberculosis* in human dendritic cells. J Immunol. 2003; 170: 1939–1948. 10.4049/jimmunol.170.4.1939 12574362

[40] Desjardins M. Biogenesis of phagolysosomes: the “kiss and run” hypothesis. Trends Cell Biol. 1995; 5:183–186. 14731444 10.1016/s0962-8924(00)88989-8

[41] Vieira OV., Botelho RJ. and Grinstein S. Phagosome maturation: aging gracefully. Biochem J. 2002; 366: 689–704. 10.1042/BJ20020691 12061891 PMC1222826

[42] Nunes P., Demaurex N., Dinauer MC. Regulation of the NADPH oxidase and associated ion fluxes during phagocytosis. Traffic. 2013; 14:1118–1131. 10.1111/tra.12115 23980663

[43] West AP., Brodsky IE., Rahner C., Woo DK., Erdjument-Bromage H., Tempst P. et al TLR signalling augments macrophage bactericidal activity through mitochondrial ROS. Nature. 2011; 472:476–480. 10.1038/nature09973 21525932 PMC3460538

[44] Nunes P., Demaurex N. The role of calcium signaling in phagocytosis. J Leukoc Biol. 2010; 88:57–68. 10.1189/jlb.0110028 20400677

[45] Mantegazza AR., Savina A., Vermeulen M., Pérez L., Geffner J., Hermine O. et al NADPH oxidase controls phagosomal pH and antigen cross-presentation in human dendritic cells. Blood. 2008; 112:4712–4722. 10.1182/blood-2008-01-134791 18682599 PMC2597138

[46] Pauwels AM., Trost M., Beyaert R., Hoffmann E. Patterns, Receptors, and Signals: Regulation of Phagosome Maturation. Trends Immunol. 2017; 38:407–422. 10.1016/j.it.2017.03.006 28416446 PMC5455985

[47] Rybicka JM., Balce DR., Chaudhuri S., Allan ERO. and Yates RM. Phagosomal proteolysis in dendritic cells is modulated by NADPH oxidase in a pH-independent manner. EMBO J. 2012; 31:932–944. 10.1038/emboj.2011.440 22157818 PMC3280544

[48] Ulrichs T., Kosmiadi GA., Trusov V., Jorg S., Pradi L., Titukhina M. et al Human tuberculous granulomas induce peripheral lymphoid follicle-like structures to orchestrate local host defence in the lung. J Pathol. 2004; 204:217–228. 10.1002/path.1628 15376257

[49] Kleinnijenhuis J., Oosting M., Joosten LAB., Netea MG. and van Crevel R. Innate Immune Recognition of Mycobacterium tuberculosis. Clin Dep Immunol. 2011; 2011:1–12. 10.1155/2011/405310 21603213 PMC3095423

[50] Greco E., Quintiliani G., Santucci MB., Serafino A., Ciccaglione AR., Marcantonio C. et al Janus-faced liposomes enhance antimicrobial innate immune response in *Mycobacterium tuberculosis* infection. Proc Natl Acad Sci USA. 2012; 109:E1360–1368. 10.1073/pnas.1200484109 22538807 PMC3361443

[51] Nisini R., Poerio N., Mariotti S., De Santis F., Fraziano M. The Multirole of Liposomes in Therapy and Prevention of Infectious Diseases. Front Immunol. 2018; 9:155 10.3389/fimmu.2018.00155 29459867 PMC5807682

[52] Canton J., Khezri R, Glogauer M, Grinstein S. Contrasting phagosome pH regulation and maturation in human M1 and M2 macrophages. Mol Biol Cell. 2014; 25:3330–3341. 10.1091/mbc.E14-05-0967 25165138 PMC4214780

[53] Ettorre A., Frosali S., Andreassi M., Di Stefano A. Lycopene phytocomplex, but not pure lycopene, is able to trigger apoptosis and improve the efficacy of photodynamic therapy in HL60 human leukemia cells. Exp Biol Med 2010; 235: 1114–1125. 10.1258/ebm.2010.009386 20660088

