# Supplementary figures and images for: Hydroalcoholic extract from Origanum vulgare induces a combined anti-mycobacterial and anti-inflammatory response in innate immune cells
Source: PLoS One. 2019 Mar 4;14(3):e0213150. doi: 10.1371/journal.pone.0213150 (PMC6398838; doi:10.1371/journal.pone.0213150)

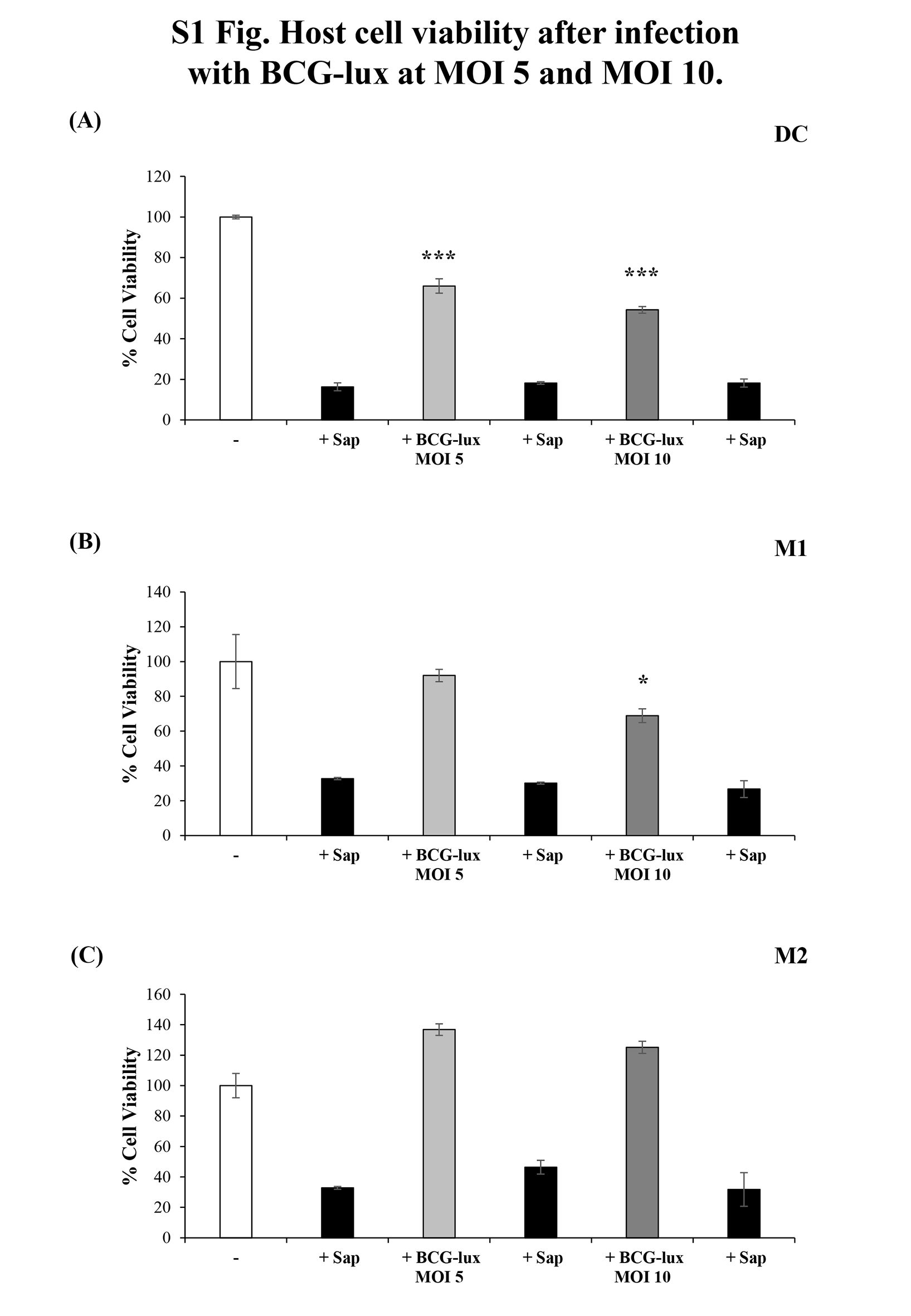

Supplement: S1 Fig — DC (A), M1 (B) and M2 (C) (2x10^5^/well) were infected with BCG-lux (MOI 5 and 10) for 3 hours, were incubated for 3 days at 37°C with 5% CO2 and were subjected to MTT assay. Data are expressed as means ± SD of % of cell viability of triplicate cultures and are representative of 2 independent experiments performed on cells from different donor. *p<0.05 and ***p<0.0001 in comparison with uninfected cells. (TIF) [file pone.0213150.s001.tif]

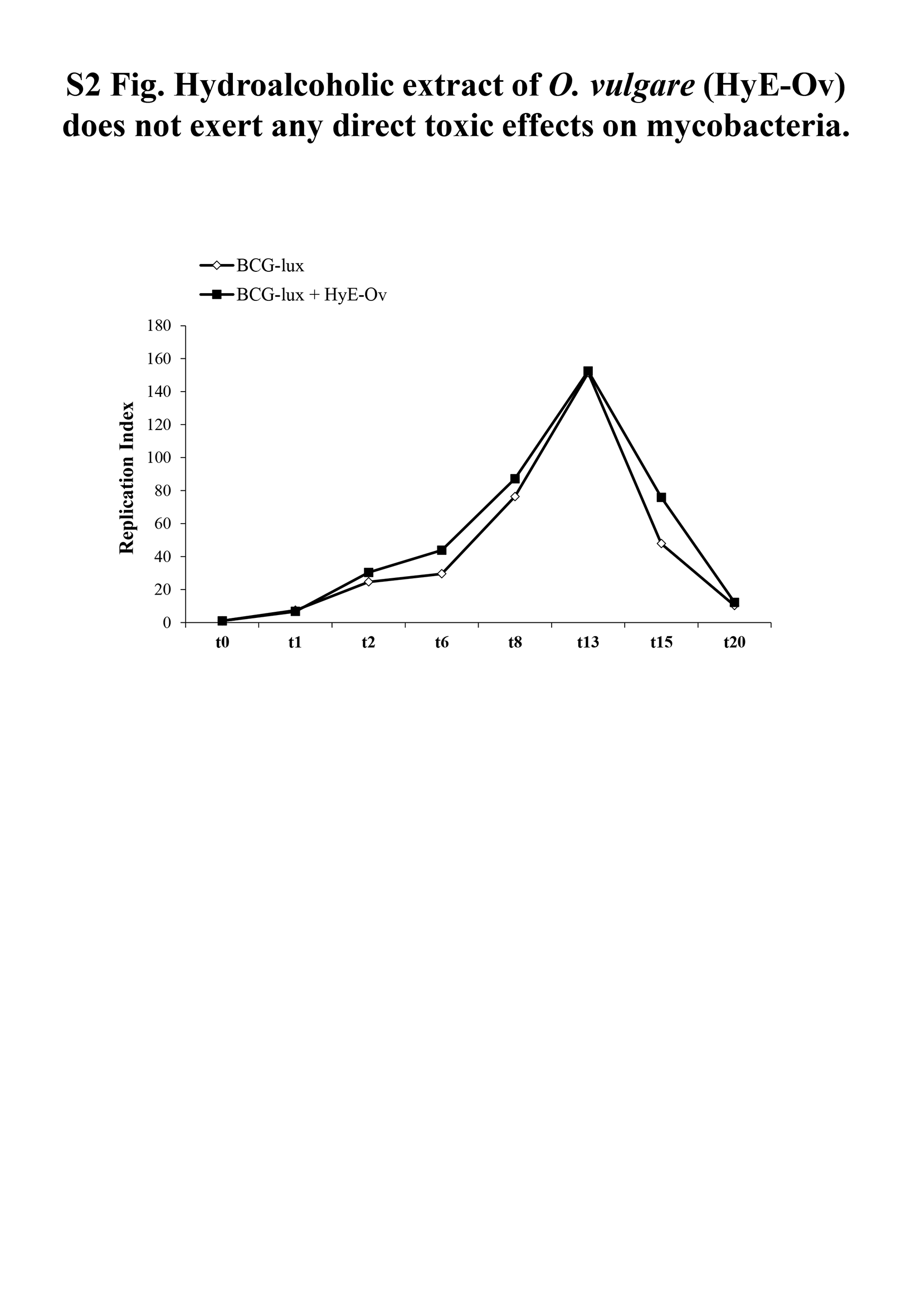

Supplement: S2 Fig — BCG-lux was cultured in 7H9 medium in the presence or absence of the extract and the mycobacterial growth was monitored for 2 weeks by a luminometric assay. Data are expressed as Replication Index, calculated as the ratio between the Relative Luminescence Units (RLU) obtained at the day indicated in figure and the RLU value obtained at the beginning of culture (t0). Data are representative of 2 independent cultures. (TIF) [file pone.0213150.s002.tif]

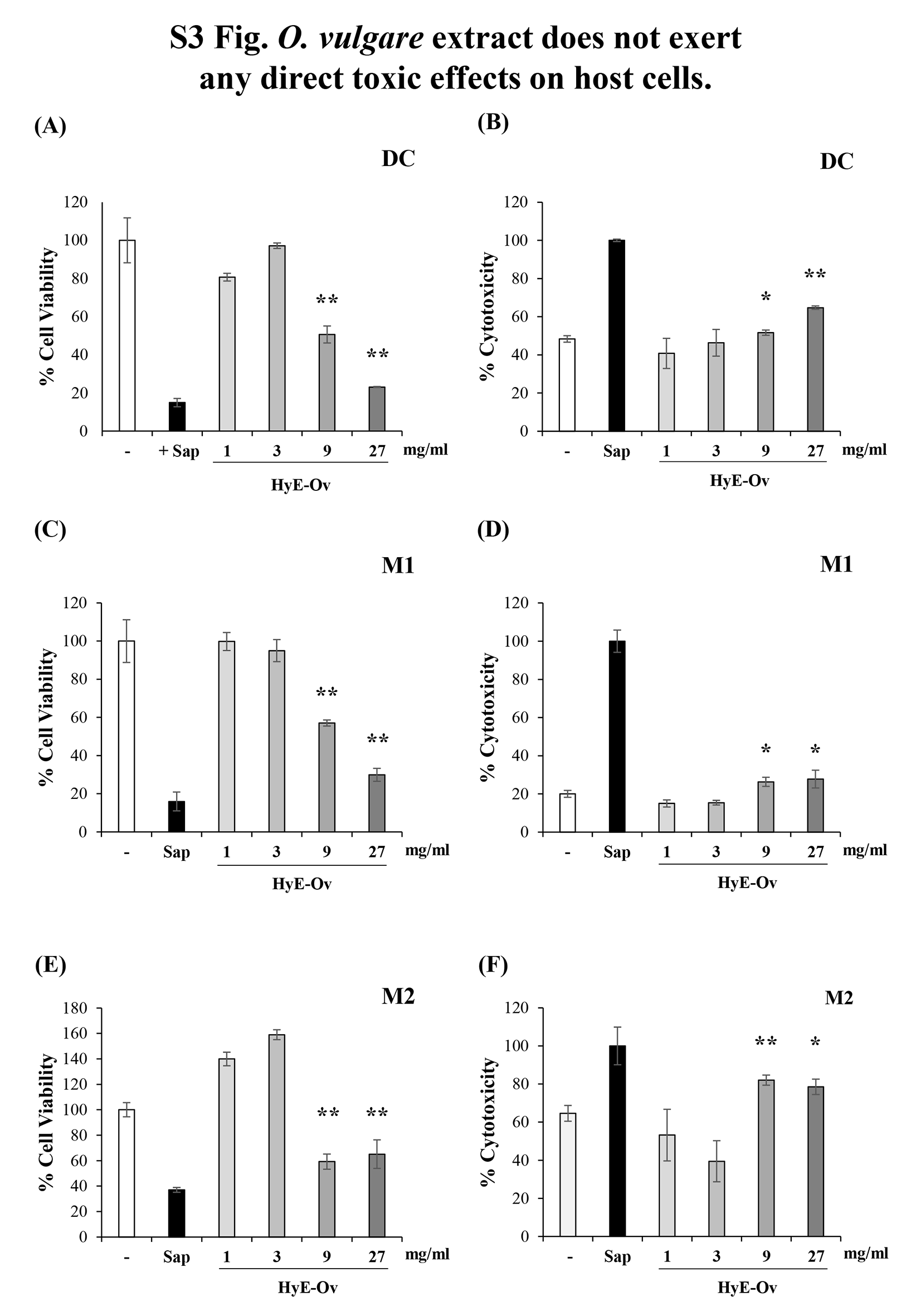

Supplement: S3 Fig — DC (A, B), M1 (C, D) and M2 (E, F) (2x10^5^/well) were stimulated with HyE-Ov at different concentration (1, 3, 9 and 27 mg/ml of equivalent plant material) for 3 days. Cells and supernatants were subjected to MTT Assay (A, C, E) and to CytoTox 96 Assay (B, D, F). Data are expressed as means ± SD of % of cell viability or % of cytotoxicity of triplicate cultures and are representative of 2 independent experiments performed on cells from different donors. *p<0.05 and **p<0.01 in comparison with non-stimulated cells. (TIF) [file pone.0213150.s003.tif]
