# Supplementary material for: Hydroalcoholic extract from Origanum vulgare induces a combined anti-mycobacterial and anti-inflammatory response in innate immune cells
Source: PLoS One. 2019 Mar 4;14(3):e0213150. doi: 10.1371/journal.pone.0213150 (PMC6398838; doi:10.1371/journal.pone.0213150)
Supplement: S1 Method — In order to evaluate the cell viability after infection, all cell types were infected with BCG-lux (MOI 5 and 10) for 3 hours. Washings were performed to block infection and to remove eventual extracellular bacteria, and cells were incubated for 3 days at 37°C with 5% CO2. MTT assay (Molecular Probe) was performed according to the manufacturer’s instructions. The MTT assay is based on the cleavage of the yellow tetrazolium salt MTT (3-(4,5-Dimethylthiazol-2-yl)-2,5-diphenyltetrazolium bromide) to purple formazan crystal in metabolically active cells. The formazan is then solubilized, and the concentration determined by optical density at 540 nm. The assay is sensitive with the colorimetric signal proportional to the viable cell number. As negative control, all cell types were treated with 0.1% saponin at 37°C for 30 min. Data are shown as means ± SD of % of cell viability of triplicate cultures. % Cell viability = 100 x Experimental OD540nm / Positive Control OD540nm. Data are representative of 2 independent experiments performed on cells from different donors. (DOCX) [file pone.0213150.s004.docx]

**S1 Method. MTT Assay.** In order to evaluate the cell viability after infection, all cell types were infected with BCG-lux (MOI 5 and 10) for 3 hours. Washings were performed to block infection and to remove eventual extracellular bacteria, and cells were incubated for 3 days at 37°C with 5% CO_2_. MTT assay (Molecular Probe) was performed according to the manufacturer’s instructions. The MTT assay is based on the cleavage of the yellow tetrazolium salt MTT (3-(4,5-Dimethylthiazol-2-yl)-2,5-diphenyltetrazolium bromide) to purple formazan crystal in metabolically active cells. The formazan is then solubilized, and the concentration determined by optical density at 540 nm. The assay is sensitive with the colorimetric signal proportional to the viable cell number. As negative control, all cell types were treated with 0.1 % saponin at 37°C for 30 min. Data are shown as means ± SD of % of cell viability of triplicate cultures. % Cell viability = 100 x Experimental OD_540nm_ / Positive Control OD_540nm_. Data are representative of 2 independent experiments performed on cells from different donors.
