# Supplementary material for: Hydroalcoholic extract from Origanum vulgare induces a combined anti-mycobacterial and anti-inflammatory response in innate immune cells
Source: PLoS One. 2019 Mar 4;14(3):e0213150. doi: 10.1371/journal.pone.0213150 (PMC6398838; doi:10.1371/journal.pone.0213150)
Supplement: S2 Method — BCG-lux was stored at -80°C and, after thawing, centrifuged and suspended in Middlebrook 7H9 (Difco) broth supplemented with 10% ADC (albumin, dextrose and catalase), 0.05% Tween 80 and 50 μg/ml Hygromycin B (Invitrogen). Bacteria were sonicated, in a bath sonicator, for 3 min to remove mycobacterial clumps and dispensed equally into two flasks, in the presence or absence of HyE-Ov (3 mg/ml of equivalent plant material). Mycobacterial growth was monitored for 2 weeks by a luminometric assay, as described [28]. Briefly, luminometric analysis was performed using PBS, mycobacterial suspension and 1% decanal (as luciferase substrate), at the ratio 8:1:1. At these experimental conditions, mycobacterial viability is directly proportional to the intensity of luminescence [30]. (DOCX) [file pone.0213150.s005.docx]

**S2 Method. Assessment of direct mycobactericidal effect of HyE-Ov**. BCG-lux was stored at -80°C and, after thawing, centrifuged and suspended in Middlebrook 7H9 (Difco) broth supplemented with 10% ADC (albumin, dextrose and catalase), 0.05% Tween 80 and 50 μg/ml Hygromycin B (Invitrogen). Bacteria were sonicated, in a bath sonicator, for 3 min to remove mycobacterial clumps and dispensed equally into two flasks, in the presence or absence of HyE-Ov (3 mg/ml of equivalent plant material). Mycobacterial growth was monitored for 2 weeks by a luminometric assay, as described [28]. Briefly, luminometric analysis was performed using PBS, mycobacterial suspension and 1% decanal (as luciferase substrate), at the ratio 8:1:1. At these experimental conditions, mycobacterial viability is directly proportional to the intensity of luminescence [30].
