# Supplementary material for: Hydroalcoholic extract from Origanum vulgare induces a combined anti-mycobacterial and anti-inflammatory response in innate immune cells
Source: PLoS One. 2019 Mar 4;14(3):e0213150. doi: 10.1371/journal.pone.0213150 (PMC6398838; doi:10.1371/journal.pone.0213150)
Supplement: S3 Method — In order to analyse the possible cytotoxic effect of HyE-Ov on dendritic cells (DC), type-1 macrophages (M1) and type-2 macrophages (M2), all cell types were stimulated with HyE-Ov at different concentrations (1, 3, 9 and 27 mg/ml of equivalent plant material). After 3 days of stimulations, cell viability was assessed by MTT assay (Molecular Probe), used according to the manufacturer’s instructions. Cells treated with 0.1% saponin at 37°C for 30 min and unstimulated cells served as a negative and positive control, respectively. Data are shown as means ± SD of % of cell viability of triplicate cultures. % Cell viability = 100 x Experimental OD540nm / Positive Control OD540nm. Data are representative of 2 independent experiments performed on cells from different donors. Simultaneously, supernatants were collected and tested by CytoTox 96 Assay (Promega) according to the manufacturer’s instructions. In particular, the CytoTox 96 Assay measures lactate dehydrogenase (LDH), a stable cytosolic enzyme that is released upon cell lysis, by means of the conversion of a tetrazolium salt (iodonitrotetrazolium violet; INT) into a red formazan product. The amount of colour formed is determined by optical density at 490 nm and is proportional to the number of lysed cells. Cells treated with 0.1% saponin at 37°C for 30 min and unstimulated cells served as a positive and negative control, respectively. Data are shown as means ± SD of % of cytotoxicity of triplicate cultures. % Cytotoxicity = 100 x Experimental OD490nm / Positive Control OD490nm. Data are representative of 2 independent experiments performed on cells from different donors. (DOCX) [file pone.0213150.s006.docx]

**S3 Method. Cytotoxicity Assays**. In order to analyse the possible cytotoxic effect of HyE-Ov on dendritic cells (DC), type-1 macrophages (M1) and type-2 macrophages (M2), all cell types were stimulated with HyE-Ov at different concentrations (1, 3, 9 and 27 mg/ml of equivalent plant material). After 3 days of stimulations, cell viability was assessed by MTT assay (Molecular Probe), used according to the manufacturer’s instructions. Cells treated with 0.1 % saponin at 37°C for 30 min and unstimulated cells served as a negative and positive control, respectively. Data are shown as means ± SD of % of cell viability of triplicate cultures. % Cell viability = 100 x Experimental OD_540nm_ / Positive Control OD_540nm._ Data are representative of 2 independent experiments performed on cells from different donors.

Simultaneously, supernatants were collected and tested by CytoTox 96 Assay (Promega) according to the manufacturer’s instructions*.* In particular, the CytoTox 96 Assay measures lactate dehydrogenase (LDH), a stable cytosolic enzyme that is released upon cell lysis, by means of the conversion of a tetrazolium salt (iodonitrotetrazolium violet; INT) into a red formazan product. The amount of colour formed is determined by optical density at 490 nm and is proportional to the number of lysed cells. Cells treated with 0.1 % saponin at 37°C for 30 min and unstimulated cells served as a positive and negative control, respectively. Data are shown as means ± SD of % of cytotoxicity of triplicate cultures. % Cytotoxicity = 100 x Experimental OD_490nm_ / Positive Control OD_490nm._ Data are representative of 2 independent experiments performed on cells from different donors.
